# Ectopic expression of poplar gene *PsnERF138* in tobacco confers salt stress tolerance and growth advantages

**DOI:** 10.48130/FR-2021-0013

**Published:** 2021-07-29

**Authors:** Lin Liu, Zihan Cheng, Wenjing Yao, Xueyi Wang, Fenglin Jia, Boru Zhou, Tingbo Jiang

**Affiliations:** 1 State Key Laboratory of Tree Genetics and Breeding, Northeast Forestry University, 51 Hexing Road, Harbin 150040, China; 2 Co-Innovation Center for Sustainable Forestry in Southern China/Bamboo Research Institute, Nanjing Forestry University, 159 Longpan Road, Nanjing 210037, China

**Keywords:** poplar, ERF, transcription factor, transgenic tobacco, salt tolerance

## Abstract

The AP2/ERF family is one of the largest plant-specific transcription factors and plays a vital role in plant growth and stress response. In this study, *PsnERF138* was cloned from *Populus alba*×*Populus glandulosa* and transformed into tobacco using the *Agrobacterium*-mediated transformation method. PsnERF138 was localized in the nucleus through subcellular localization assay in tobacco. Under normal conditions, the root lengths of *PsnERF138* transgenic lines were much longer than those of wild type. Under salt stress, the transgenic tobacco lines over-expressing *PsnERF138* showed a significant increase in seed germination rate, plant height, and root length, compared to control plants. In addition, the transgenic tobacco lines displayed some advantages at the physiological level, such as higher superoxide dismutase (SOD) activity, peroxidase (POD) activity, proline content, and lower malondialdehyde (MDA) content, as compared to those in the control plants. Histochemical staining also showed that the transgenic tobacco lines had lower reactive oxygen species (ROS) accumulation, compared to control plants under salt stress. The combined results indicate that poplar *PsnERF138* plays a contributing role in augmenting salt tolerance and conferring multiple growth advantages as being overexpressed in tobacco.

## INTRODUCTION

Sessile plants growing under natural conditions are inevitably subjected to various environment stresses. On the contrary, abiotic stresses such as drought, salinity, and extreme temperatures have been shown to be the prevalent impetuses in accelerating plant evolution through developing various molecular mechanisms to adapt to constantly challenging environmental conditions^[1,2]^. When subjected to environmental stresses, plants perceive the changes and then transduce environmental signals via multiple signal transduction pathways, which result in activating or repressing expression of stress response and tolerance genes. Among them, soil salinization is one of the major abiotic stresses that negatively affects global agricultural production^[3]^. High salinity affects plant growth and development, resulting in reduced survival, photosynthetic rate, mineral element uptake rate and productivity^[4]^. Therefore, the study of salt tolerance has always been a research hotspot in plants, and mounting reports on identification of stress-related genes have sparked the excitement of exploration of stress tolerance genes specially transcription factors (TFs) in different plant species^[5−7]^. In the field of plant biotechnology, modulation of gene expression activity is a new direction for regulating plant growth, development, and improving plant tolerance.

About 7% of the coding sequences in plant genome are TF genes, which account for a large proportion of genes related to plant transcription regulation^[8]^. Numerous ERF TFs (Ethylene-responsive factor) have been identified in many plants such as *Arabidopsis thaliana*, rice, corn, cucumber, soybean, poplar, etc.^[9−12]^. The TFs functionally related to drought, high salt, low temperature, hormones, pathogenic response and growth and development have been identified in higher plants^[13]^, such as bZIP, NAC,WRKY, MYC/MYB and AP2/ERF^[14−16]^. The plant-specific family, AP2/ERF, is one of the largest TF families in plants, accounting for approximately 9% of the known plant TF genes, and plays an important role in plant response to different stresses^[16−19]^. The AP2/ERF family has been divided into five sub-families that include AP2, ERF (Ethylene-responsive factor), DREB (Dehydration-responsive element binding proteins), RAV (Related to ABI3/VP1) and Soloist^[20]^.

Some ERFs have been shown to be involved in a series of important growth processes in plants, including hormone signal induction, metabolic regulation, cell differentiation, plant growth and development^[21−23]^. Some other ERFs have been shown to respond to abiotic stresses such as drought and salinity. Overexpression of these genes improved stress tolerance in transgenic plants. For example, *Arabidopsis thalian ERF1* gene responds to drought and high salt stress by binding to GCC-box and DRE elements of target genes^[24]^. Overexpression of the *OPBP1* gene increases salt tolerance of transgenic tobacco lines^[25]^. Overexpression of *TaERF3* gene improves drought and salt tolerance of transgenic plants^[26]^. Our previous studies also indicate that ERF family members play an important role in the response to multiple abiotic stresses^[27]^. For example, transgenic poplars overexpressing the *PthERF168* gene displayed higher salt tolerance, as compared to wild type (WT) plants^[28]^. In addition, we also found that poplar *PsnERF138 *is highly induced by multiple abiotic stresses^[29]^. To advance our understanding of the biological functions of *PsnERF138* in salt tolerance, we cloned the gene from poplar and transformed it into tobacco. The roles of *PsnERF138* in salt stress response and tolerance were substantiated by characterizing the stress-determining traits of transgenic tobacco lines at morphological, physiological and histochemical levels under salt stress treatment. We revealed that *PsnERF138* could confer many growth advantages to transgenic plants under salt stress conditions.

## RESULTS

### Subcellular localization of PsnERF138

**Figure 1:**
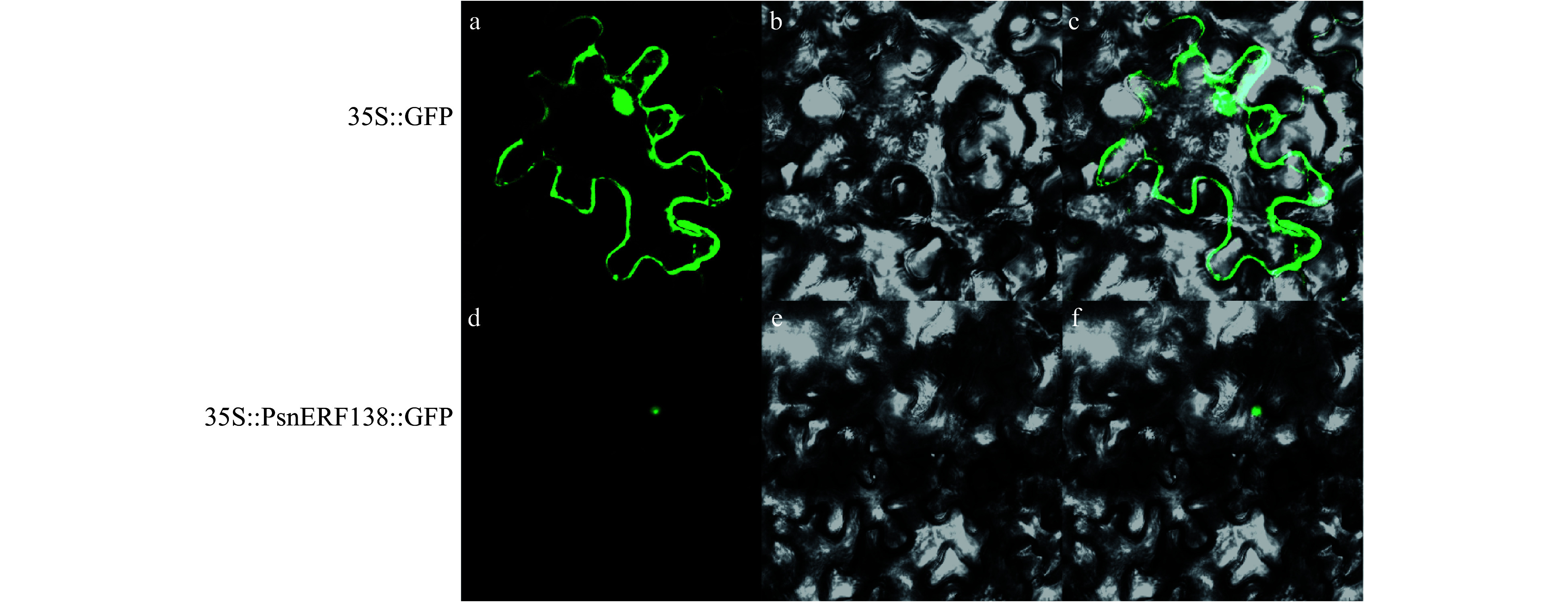
Localization of PsnERF138 in the nucleus. 35S::GFP (a−c) and 35S::PsnERF138::GFP (d−f) were injected into tobacco epidermal cells. (a) and (d) showed green fluorescence in dark field; (b) and (e) were observed in the bright field; (c) and (f) were the mixed observation of light and dark fields.

The fluorescent signals in transgenic tobacco were observed by confocal laser scanning microscopy (Fig. 1). It showed that fluorescent signals in the leaves of 35S::PsnERF138-GFP transgenic lines were detected in the nuclei, while no fluorescent signals were observed in whole cells in the control lines containing 35S-GFP. The results indicate that PsnERF138 was located in the nuclei.

### Molecular validation of transgenic tobacco

**Figure 2:**
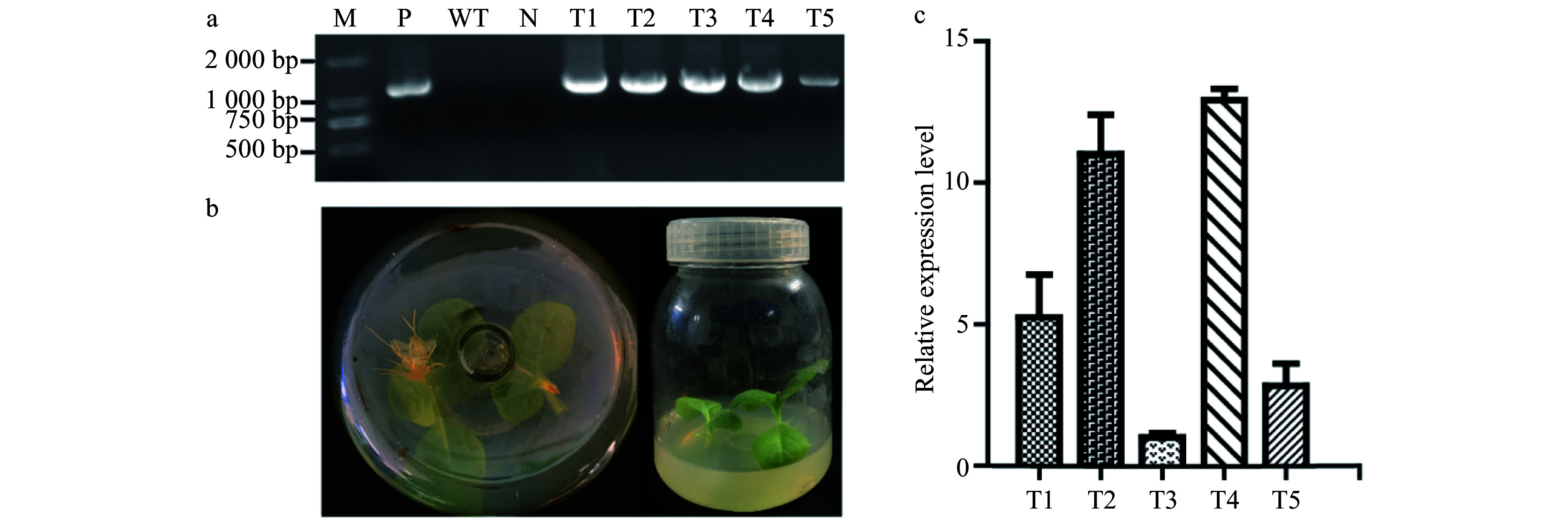
Identification of genetically transgenic tobacco. (a) PCR detection of the transgenic lines; M, 2000 DNA maker; P, positive control with recombined vector as the template; N, negative control using water as a template; WT, wildtype tobacco; T1−T5, transgenic tobacco lines. (b) Transgenic lines could be rooted in rooting medium containing 100 mg/ml kanamycin. Wildtype tobaccois on the bottom and grew well. (c) Expression of *PsnERF138* in five transgenic lines and wildtype tobacco. Each error bar represents the standard deviation of three independent replicates.

Five transgenic lines were obtained by *A. tumefaciens*-mediated gene transformation. They grew normally in the medium containing 100 ml/L Kan (Fig. 2b). Genomic DNA was extracted from the transgenic lines and wild type (WT) plants. The expected bands of *PsnERF138* were detected in the transgenic lines, which were consistent with the positive control. As expected, there was no bands amplified in both the WT plant and pure water control (Fig. 2a). The expression levels of *PsnERF138* in five transgenic tobacco lines were different, among which the expression levels of T1, T2 and T4 werenhigher than the remaining two lines (Fig. 2c), and thus were used for salt tolerance analysis.

### Salt tolerance tests of transgenic tobacco

**Figure 3:**
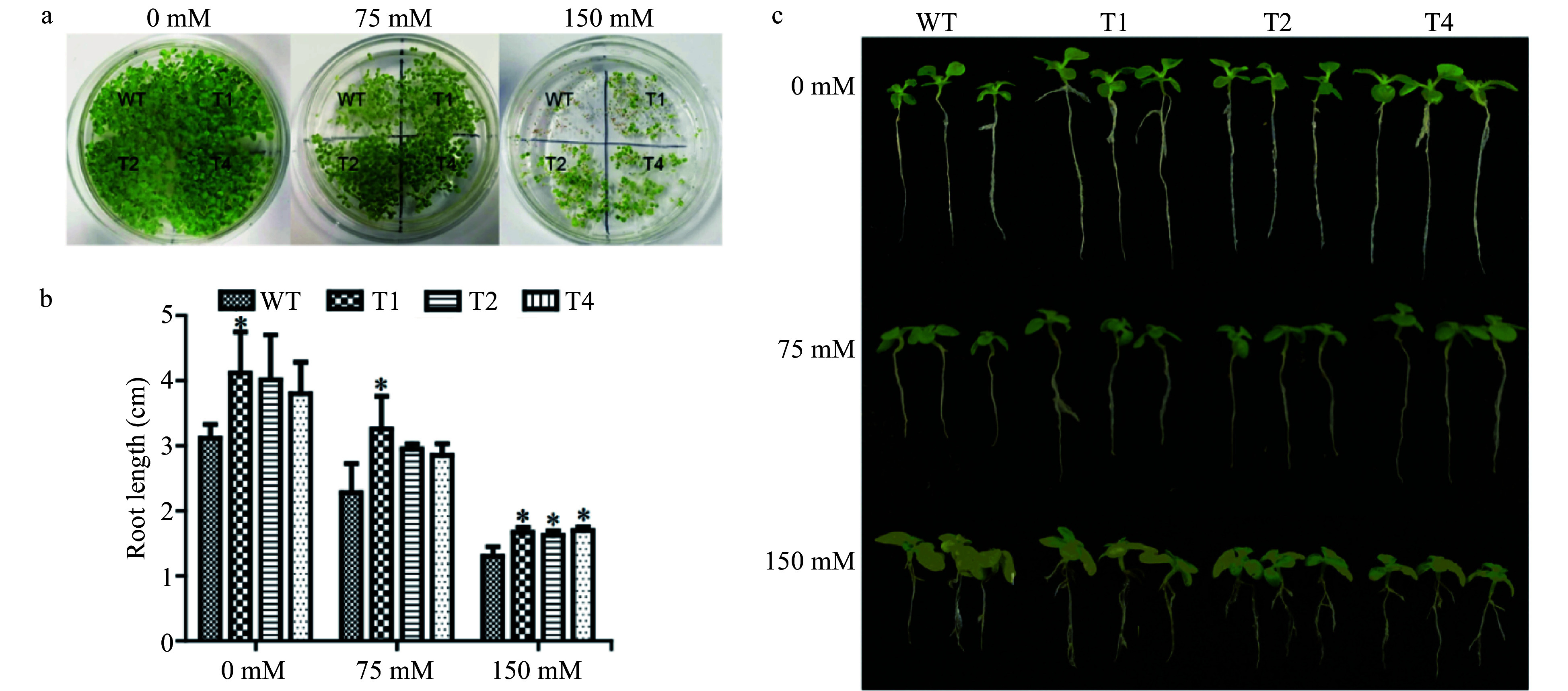
Germination and growth of transgenic tobacco and WT tobacco and their phenotypes under salt stress. (a) Germination and growth of transgenic tobacco and WT tobacco, T_1_, T_2_ and T_4_ transgenic tobacco lines. (b) Seedlings moved to 0, 75 and 150 mM NaCl to record root length data. (c) Phenotype of transgenic and WT tobacco seedlings under salt stress. Each error bar represents the standard deviation of three independent replicates, and each asterisk indicates that there was a significant difference between a transgenic line and wild type as tested with Analysis of Variance (ANOVA) with *p* < 0.05.

There was no noticeable difference in the germination rates between the transgenic lines and WT plants under normal conditions. However, the average germination rate of the three transgenic lines was 1.26 ± 0.14 and 3.57 ± 1.71 higher than those of WT plants, under 75 and 150 mM NaCl stress, respectively (Fig. 3a). In addition, the root lengths of all the transgenic lines were longer than those of WT plants under both control and salt stress conditions (Fig. 3c). Under normal conditions, the root lengths of transgenic lines were 1.22−1.31 times longer than that of WT plants. Under 75 mM NaCl stress, the root lengths of transgenic tobacco were 1.25−1.43 times longer than that of WT plants, and under 150 mM NaCl stress, the root lengths of the transgenic tobacco were 1.24−1.31 times longer than that of the WT (Fig. 3b, c). These results showed that overexpression of *PsnERF138* conferred some growth advantages to the transgenic lines.

**Figure 4:**
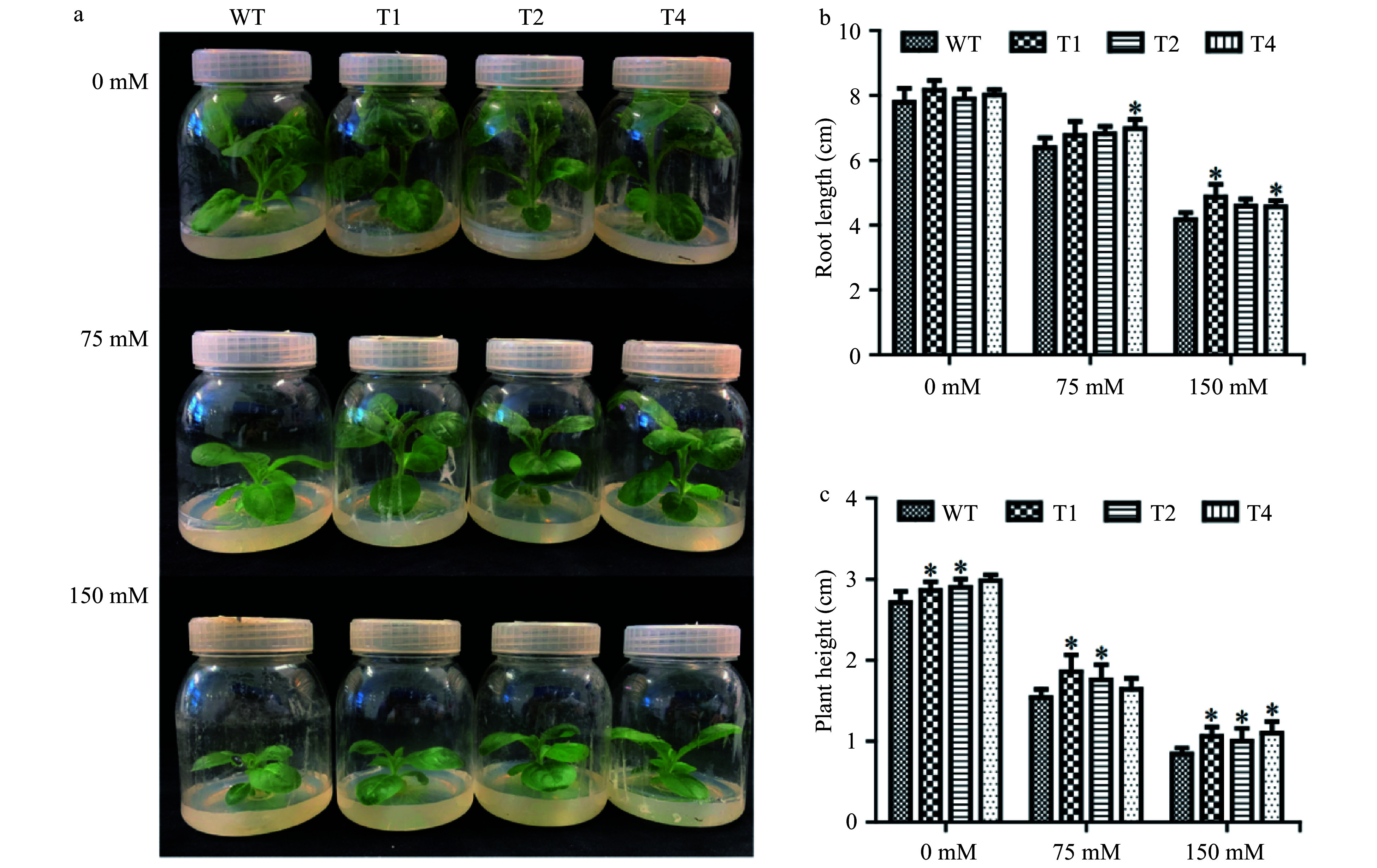
Growth of transgenic tobacco under salt stress. (a) Comparison of growth of WT and transgenic lines under 0, 75, 150 mM NACL salt stress. (b) Plant heights of WT and transgenic lines. (c) Root lengths of WT and transgenic lines. Each error bar represents the standard deviation of three independent replicates, and each asterisk indicates that there was a significant difference between a transgenic line and wild type as tested with Analysis of Variance (ANOVA) with *p* < 0.05.

The plant heights and root lengths of the transgenic lines and WT plants were also determined with 0, 75 and 150 mM NaCl treatments for one month in tissue culture bottles. Under control conditions, the plant heights of tobacco transgenic lines were not significantly different from that of WT plants. Under 75 and 150 mM NaCl stress conditions, the plant heights of the transgenic lines were higher than that of the WT plant (Fig. 4a). Under control conditions, the plant height and root length of the transgenic plants were 1.05−1.1 times higher and 1.02−1.05 times longer than those of WT plants, respectively, but the differences were not beyond the significant levels. Under 75 mM salt stress, the plant heights and root lengths of the transgenic plants were 1.06−1.2 times higher and were 1.06−1.7 times longer than those of WT plants, respectively. Under 150mM salt stress, the plant height and root length of the transgenic plants were 1.19−1.3 times higher and 1.09−1.16 times longer than those of WT plants, respectively (Fig. 4b). These results imply that transgenic tobacco lines had morphological growth advantages compared to WT under salt stress conditions.

### Physiological analysis of transgenic tobacco lines overexpressing *PsnERF138*

**Figure 5:**
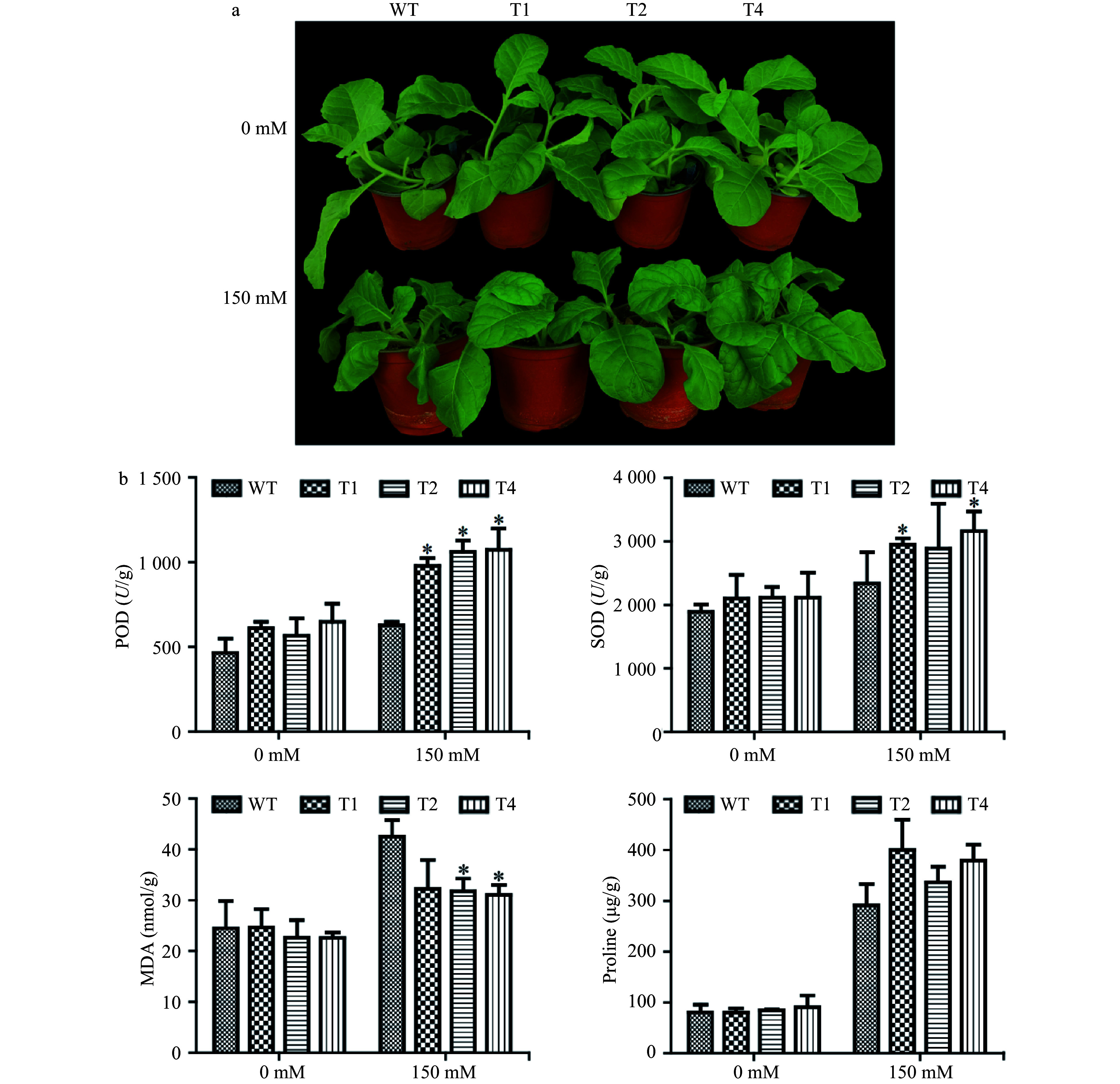
Physiological analyses of WT and transgenic tobacco. (a) Growth comparison of transgenic tobacco and WT in soil under salt stress. (b) The physiological parameters include superoxide dismutase (SOD) activity, peroxidase (POD) activity, malondialdehyde (MDA) and proline content. The transgenic lines and wild type were compared under 0 and 150 mM NaCl conditions respectively. Each error bar represents the standard deviation of three independent replicates, and each asterisk indicates that there was a significant difference between a transgenic line and wild type as tested with Analysis of Variance (ANOVA) with *p* < 0.05.

To further test salt tolerance of *PsnERF138* overexpression tobacco transgenic lines under natural conditions, the 30-day-old seedlings were irrigated with 150 mM NaCl solution for 15 days, the WT leaves were wilted, whereas the transgenic lines grew well (Fig. 5a), indicating that the transgenic plants ectopically overexpressing *PsnERF138* had gained significant salt tolerance, as compared to WT.

Under normal conditions, the superoxide dismutase (SOD) and peroxidase (POD) activity of the transgenic lines were 1.1 ± 0.1 and 1.21 ± 0.18 times higher than those of the WT plants, respectively (Fig. 5b). However, there was no significant difference between the transgenic lines and WT plants in MDA and proline content. Under 150 mM NaCl treatment, the POD activity, SOD activity, and proline content were 1.23 ± 0.12, 1.15 ± 0.22, and 1.56 ± 0.15 of those in WT, respectively. Only the MDA content in the WT was1.31 ± 0.03 times higher than the average value of three transgenic lines (Fig. 5b). These lines of evidence indicate that ectopically expressing *PsnERF138* transgenic plants conferred better salt tolerance than wild type.

### Biochemical and physiological changes by histochemical staining analysis

**Figure 6:**
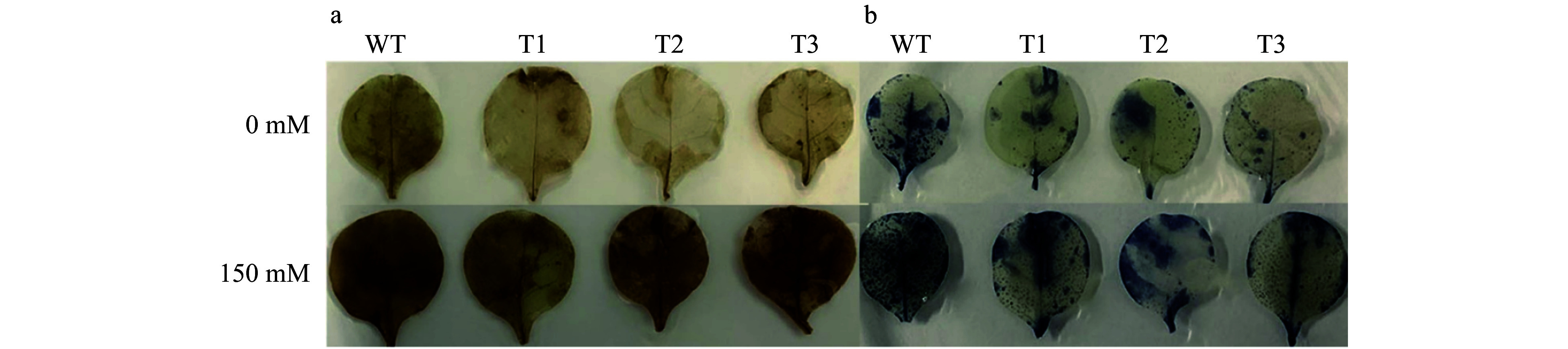
(a) Histochemical staining with 3, 3’-diaminobenzidine (DAB). (b) Histochemical staining with nitrotetrazolium blue chloride (NBT).

To determine if the salt tolerance of *PsnERF138* overexpression lines was conferred by biochemical and physiological changes, we measured reactive oxygen species in wild-type and transgenic lines under control and cold salt treatment conditions. DAB could be oxidized by H_2_O_2_ in the presence of peroxidase to produce a red-brown precipitate, and NBT can react with superoxide radicals ($\text O_2^- $) to form a dark blue insoluble formaldehyde compound^[30,31]^. Under normal conditions, the color depths of the transgenic lines and WT plants in leaves were similar with DAB and NBT stainings. However, under salt stress, DAB and NBT staining showed that the leaf colors of WT plants were significantly darker than these of transgenic plants (Fig. 6). The results proved that reactive oxygen species (ROS) accumulation in WT plants were higher than those in the transgenic lines, indicating that the transgenic lines had higher ROS scavenging ability than the WT.

## DISCUSSION

Under harsh environmental stresses, sessile plants develop complex mechanisms to adapt to the adversity at the molecular level, these defense mechanisms involve a variety of signaling pathways, including abscisic acid, jasmonic acid, ethylene, salicylic acid and other signaling pathways. Transcription factors play a key role in activating or inhibiting the expression of defense genes in these signaling pathways. In addition, transcription factors also regulate the interactions between different signaling pathways so that plants can better adapt to the adverse environment and improve plant resistance^[32−35]^. In recent years, some AP2/ERF transcription factor family members have been found to play important roles in stress response and tolerance and also growth. Although ERF subfamily proteins, which are generally located downstream of the ethylene signal transduction pathway, can regulate ethylene biosynthesis and signal transduction in a feedback loop. Through signal transduction, ethylene induces ERFs to regulate stress defense, growth and development in plants^[7,36]^.

In the process of plant evolution, due to the whole genome, fragment doubling, and chromosome exchange and the need to adapt to various environmental conditions, the AP2/ERF gene family expended significantly. Under stress condition, the neofunctionalization of duplicated genes in sessile plants could be reinforced^[11]^. To date, the ERFs genes have become the largest TF family in plants, and can be classified into two large categories: (1) those that can enhance one gene for multiple stresses. For example, ectopic expression of the *GmERF3* gene confers enhanced tolerance to high salinity and dehydration stresses in transgenic tobacco^[37]^. Transgenic lines overexpressing *AtERF1* are more tolerant to drought and salt stress^[24]^. The ERF transcription factor *TaERF3* enhances tolerance to salt and drought stresses in wheat^[26]^. Overexpression of *BplERF1* and *BplERF13* in birch can enhance its cold tolerance^[38,39]^. The *EIN2* gene is required for lead resistance in *Arabidopsis*^[40]^. Overexpression of *LchERF* in tobacco enhances its tolerance to cadmium stress^[41]^. (2) Those that can not only enhance stress response and tolerance, but also promote growth. For example, overexpression of *HbERF-IXc5* enhances the tolerance of *Hevea brasiliensis* to water deficit, cold and salt stresses, and promotes the development of its root system^[42]^. In addition, overexpression of the *PsnERF76* gene in poplar resulted in increased root length and plant height, and improved salt tolerance^[43]^. Moreover, overexpression of *OsERF48* can enhance root growth and drought tolerance in rice through regulating *OsCML16c*^[44]^. Finally, overexpression of the *PsnERF138* gene not only enhances salt tolerance, but also results in growth advantage under salt stress.

Why can the ERFs in the secondary category promote growth under stress conditions? We believe there are several possibilities: (1) these ERF proteins are involved in a positive feedback mechanism to increase ethylene synthesis, which can in turn promote growth. For example, overexpression of the *ERF76* gene in poplar regulates the expression of ethylene synthesis related genes, resulting in the enhancement of salt tolerance^[43]^. Tomato *LeERF2* is an important regulator of ethylene biosynthesis gene expression and ethylene production, and overexpression of *LeERF2* in tomato can improve its cold tolerance^[45−47]^ and results in longer roots than seen in WT. *OsERF109* negatively regulates ethylene biosynthesis and drought tolerance in rice^[48]^. *OsERF3* negatively affected ethylene production and drought tolerance in rice^[49]^; (2) these ERFs may directly regulate growth pathways. For example, *ERF34*, *ERF35*, *ERF38*, and *ERF39* genes are expressed in regions related to cell division or cell differentiation in seedlings and flowers to participate in the development process^[50]^. *Arabidopsis ERF115* controls root quiescent center cell division and stem cell replenishment^[51]^. Poplar *ERF139* coordinates xylem cell expansion and secondary cell wall deposition^[52]^; (3) ERFs remove ROS so efficiently that the ROS in transgenic lines are even lower than those in WT, thereby enhancing growth. For example, *MbERF11* may play an important role in the response of *Arabidopsis* to cold and salt stresses by enhancing the scavenging ability of ROS^[53]^. Trifoliate orange *PtrERF109* maintains strong antioxidant capacity by directly regulating the POD-encoding genes, thereby conferring cold tolerance to plants^[54]^. *Tamarix hispida* ERF transcription factor gene *ThCRF1* can regulate the ability of ROS scavenging to improve plant salt tolerance^[55]^. Our *PsnERF138* appears to promote ethylene biosynthesis, however, further studies are needed to clarify this.

How ERFs promote the stress response and tolerance remains unknown. We do however know that ERF proteins can regulate some stress tolerance genes. For example *BplERF1* regulates *WRKY70*^[38]^. In addition, we know plants have evolved adaptive molecular mechanisms to respond to salt stress through a series of physiological changes^[56]^. Plants produce osmotic adjustment compounds in response to salt stresses, such as proline^[57,58]^. High proline content is beneficial for maintaining plant cell structure and function, thereby improving plant salt tolerance^[59,60]^. Previous studies showed that transgenic plants with higher proline levels increase tolerance to salt and osmotic stress^[61,62]^. Our study showed that *PsnERF138* overexpressing transgenic tobacco lines produced more proline than WT. Therefore, *PsnERF138* appears to serve as a positive regulator in salt stress responses by biosynthesis of osmoprotectants.

Salt stress affects the permeability of plant cell membranes and increases the accumulation of ROS, ROS can aggravate the destruction of proteins, lipids, carbohydrates and DNA, eventually leading to lipid peroxidation and accelerating aging^[63,64]^. POD and SOD are two important antioxidant enzymes, which can reduce H_2_O_2_ and ${\text O}^-_2 $^[65]^, respectively, and thereby improving salt tolerance of plants^[66−68]^. MDA, as the product of lipid peroxidation, is an indicator of damage of the plant cell membrane^[69,70]^. In our study, *PsnERF138* lines showed increased POD and SOD activities, and reduced MDA, H_2_O_2_ and ${\text O}^-_2  $, which was similar to that observed in the transgenic *Betula platyphylla* transgenic lines overexpressing *BplERF13*^[39]^ and *BplERF1*^[38]^, in which the decrease of reactive oxygen species significantly improves cold stress tolerance. Tomato *SIERF5* overexpressing transgenic lines with increases in salt tolerance also exhibit augmented ROS scavenging ability^[71]^. These results indicate that overexpression of ERF genes eliminates the ROS generated in the stress response.

For those ERFs that can not only enhance stress tolerance but also bring some growth advantages under stress conditions, future research should focus on the underlying molecular mechanisms. We should identify their downstream target genes to explain how growth advantages are generated in addition to stress tolerance.

## CONCLUSIONS

In summary, under salt stress, transgenic tobacco lines overexpressing *PsnERF138* displayed morphological, physiological and biochemical advantages as compared to WT. At the biochemical and physiological levels, the POD and SOD activities and proline content in the transgenic lines were higher than those in WT, whereas the MDA content was significantly reduced. The DAB and NBT staining indicated less ROS were accumulated in the *PsnERF138* transgenic lines than WT. *PsnERF138* transgenic lines manifested many growth advantages compared to WT plants. Seed germination rates, and plant heights of the *PsnERF138* transgenic tobacco lines were higher and the root lengths were much longer than those of WT plants under salt stress. The above results indicate that overexpression of *PsnERF138* could enhance salt tolerance of transgenic tobacco by increasing POD, SOD activities and proline content which eliminates ROS accumulation. Future research should examine if *PsnERF138* can promote growth by regulating ethylene biosynthesis. This study provides valuable evidence that the use of *PsnERF138* can enhance salt stress tolerance of plants without reducing growth and development.

## MATERIALS AND METHODS

### Plant materials and growth conditions

To prepare the explants for transformation, the WT tobacco (*Nicotiana tabacum* L. cv. Petit Havana SR-1) seeds were immersed in NaClO solution (1% Cl, 0.05% TWEEN20) for 10 min and washed with sterilized water five times. The seeds were then sown on horizontal plates containing MS medium with 30 g/L sucrose under 23 ± 2 °C and 16/8-h light/dark for one week. Healthy tobacco seedlings were transferred into tissue culture bottles containing Murashige and Skoog (MS) medium^[72]^.

The leaves of tobacco seedlings grown in MS medium were cut to 1 cm × 1 cm dimension, which were then used directly as recipients of *Agrobacterium tumefaciens* infection. Meanwhile, wild type seedlings of 84 K poplar (*Populus alba *× *Populus glandulosa*) were cultured at 23 ± 2 °C and 16/8-h light/dark for 30 days. The shoots from the same clone of 84 K poplar were hydroponiced. After growth of a few new roots was seen, RNA was extracted and stored at –80 °C for gene cloning.

### Gene cloning and vector construction

Fresh leaves from 84K poplar seedlings were collected and frozen in liquid nitrogen for RNA isolation. Total RNA was extracted using RNA Extraction Kit (Takara, China). The cDNA was obtained using Prime Script^TM^ RT reagent Kit with gDNA Eraser (Takara, Dalian, China).

The full sequence of *PsnERF138’s counterpart*in *P. trichocarpa*, Potri.006G138900.1, was queried from the *Populus trichocarpa* v3.0 database in Phytozome12 (https://phytozome.jgi.doe.gov/pz/portal.html). We designed a pair of primers from Potri.006G138900.1 using a primer design tool called Bioxm 2.6, and obtained a pair of primers (ERF-F1: ATGGTGAGAGAGAGAAGGGAGAGA; ERF-R1: CACGGGACAGTAAAGAAGAAGAAG), which were used to amplify the transcript sequence of *PsnERF138* from 84K gene using RT-PCR. The PCR products were cloned into pBI121 vector to construct the recombinant vector 35S-PsnERF138.

### Subcellular localization of PsnERF138 protein

The coding region of *PsnERF138* without termination codon was fused into pBI121 vector to construct 35S::PsnERF138::GFP vector. The plasmid 35S::PsnERF138::GFP and *35S-GFP *(control) were transfected into tobacco and cultured in Luria-Bertani (LB) liquid medium (containing kanamycin and rifampicin) until OD_600_ = 1.0. The recombinant plasmid containing *PsnERF138-GFP* was injected into the tobacco epidermis by needle injection. The expression site of PsnERF138-GFP fusion protein in tobacco cells was examined by laser confocal microscopy (LSM 700, Zeiss, Germany).

### Gene transformation and identification of transgenic tobacco lines

The PsnERF138::GFP Agrobacterium solution was absorbed into 100 ml LB liquid medium containing 50 mg/L rifampin and 50 mg/L kanamycin. The shaking bed was incubated at 28 °C and was kept in the range of 0.6−0.8 at 200 rpm. The tobacco leaves were immersed in the bacterial solution for 10 min. The leaf pieces were then transferred to differentiation medium 2−4 days in the dark. Tobacco leaf pieces were then transferred into the selective medium containing 100 mg/L kanamycin and 200 mg/L ceftriaxone sodium, and cultured under light in the tissue culture room; when the leaves differentiated resistant buds, they were cut off and transferred to rooting medium containing 100 mg/L kanamycin and 200 mg/L ceftriaxone sodium^[73]^. Transgenic tobacco seedlings were selected in the MS medium containing 100 mg/L Kan. Furthermore, DNA was extracted from WT and the transgenic tobacco leaves and then RT-PCR was conducted with primers ERF-F2 and ERF-R2 (ERF-F2: CCATCGTTGAAGATGCCTCTGC; ERF-R2: CTCTTCGCTATTACGCCAGCTG). The T_2_ transgenic lines which presented a 3:1 segregation ratio by Kan screening were selected for further analysis. A total of five transgenic tobacco lines in T_3_ generation were selected for quantitative analysis of gene expression.

### Characterization of growth traits of transgenic tobacco lines

One hundred seeds from each transgenic line and WT were placed on MS medium supplemented with 0, 75 and 150 mM NaCl, respectively, to obtain the germination rate. The transgenic lines and WT with similar size upon growing in MS medium for 7 days were transplanted into MS medium containing 150 mM NaCl for 7 days, and the root lengths were measured.

Transgenic seedlings at the two leaf stage were transferred into MS medium containing 0, 75 and 150 mM NaCl, respectively. After one month, the plant heights and root lengths of the seedlings were measured. To apply the salt treatment, the seedlings cultivated in the soil for two weeks in a greenhouse were irrigated with 0 and 150 mM NaCl solution respectively for 15 days. Three independent experiments with three biological replicates were performed for each measurement.

### Physiological trait measurement and analysis

A few physiological indexes related to stress response including SOD activity, POD activity, MDA content and proline content in transgenic lines and WT plants of approximately 2 months in age were determined under salt stress. All the experiment kits used for physiological analysis were from Nanjing Jiancheng Institute of Biological Engineering (Nanjing, China).

### Biochemical and physiological changes

The detection of hydrogen peroxide (H_2_O_2_) and superoxide ($\text O_2^- $) anion in the transgenic lines and WT under salt stress were carried out by histochemical staining with diaminobenzidine (DAB) and nitrotetrazolium blue chloride (NBT) as chromogenic substrates, respectively^[31]^.

### Statistical analysis

We used SPSS version 22 (Chicago, IL, USA) to analyze the standard deviation of the experimental data. The statistical significance level was set to a *p*-value ≤ 0.05. If the value ≤ 0.05, the difference was considered to be significant. The data were presented as mean ± standard error (SE) with each SE being calculated from three independent biological samples.
